# Cardiovascular disease by diabetes status in five ethnic minority groups compared to ethnic Norwegians

**DOI:** 10.1186/1471-2458-11-554

**Published:** 2011-07-13

**Authors:** Anh T Tran, Jørund Straand, Lien M Diep, Haakon E Meyer, Kåre I Birkeland, Anne K Jenum

**Affiliations:** 1 Department of General Practice, Institute of Health and Society, University of Oslo, Oslo, Norway; 2 Department of Biostatistics and Epidemiology, Oslo University Hospital, Ullevål, Oslo, Norway; 3 Department of Community Medicine, Institute of Health and Society, University of Oslo, Oslo, Norway; 4 National Centre for Suicide Research and Prevention, Institute for Clinical Medicine, University of Oslo, Oslo, Norway; 5 Division of Epidemiology, Norwegian Institute of Public Health, Oslo, Norway; 6 Diabetes Research Centre, Faculty of Medicine, Oslo University Hospital, Aker, Oslo, Norway; 7 Faculty of Health Sciences, Oslo University College, Oslo, Norway

**Keywords:** Cardiovascular disease, diabetes, ethnicity, socioeconomic position, risk factors.

## Abstract

**Background:**

The population in Norway has become multi-ethnic due to migration from Asia and Africa over the recent decades. The aim of the present study was to explore differences in the self-reported prevalence of cardiovascular disease (CVD) and associated risk factors by diabetes status in five ethnic minority groups compared to ethnic Norwegians.

**Methods:**

Pooled data from three population-based cross-sectional studies conducted in Oslo between 2000 and 2002 was used. Of 54,473 invited individuals 24,749 (45.4%) participated. The participants self-reported health status, underwent a clinical examination and blood samples were drawn. A total of 17,854 individuals aged 30 to 61 years born in Norway, Sri-Lanka, Pakistan, Iran, Vietnam or Turkey were included in the study. Chi-square tests, one-way ANOVAs, ANCOVAs, multiple and logistic regression were used.

**Results:**

Age- and gender-standardized prevalence of self-reported CVD varied between 5.8% and 8.2% for the ethnic minority groups, compared to 2.9% among ethnic Norwegians (p < 0.001). Prevalence of self-reported diabetes varied from 3.0% to 15.0% for the ethnic minority groups versus 1.8% for ethnic Norwegians (p < 0.001). Among individuals without diabetes, the CVD prevalence was 6.0% versus 2.6% for ethnic minorities and Norwegians, respectively (p < 0.001). Corresponding CVD prevalence rates among individuals with diabetes were 15.3% vs. 12.6% (p = 0.364). For individuals without diabetes, the odds ratio (OR) for CVD in the ethnic minority groups remained significantly higher (range 1.5-2.6) than ethnic Norwegians (p < 0.05), after adjustment for age, gender, education, employment, and body height, except for Turkish individuals. Regardless of diabetes status, obesity and physical inactivity were prevalent in the majority of ethnic minority groups, whereas systolic- and diastolic- blood pressures were higher in Norwegians. In nearly all ethnic groups, individuals with diabetes had higher triglycerides, waist-to-hip ratio (WHR), and body mass index compared to individuals without diabetes. Age, diabetes, hypertension, hypercholesterolemia, and WHR were significant predictors of CVD in both ethnic Norwegians and ethnic minorities, but significant ethnic differences were found for age, diabetes, and hypercholesterolemia.

**Conclusions:**

Ethnic differences in the prevalence of CVD were prominent for individuals without diabetes. Primary CVD prevention including identification of undiagnosed diabetes should be prioritized for ethnic minorities without known diabetes.

## Background

Over 80% of the global burden of cardiovascular disease (CVD) occurs in low- and middle-income countries, and large variations in risk factor profiles and disease rates by ethnic groups have been reported [1,2]. Migration from low- to high-income countries may lead to changes in CVD risk factors [3], and most, but not all, ethnic minority groups (EMGs) demonstrate a higher prevalence of risk factors for CVD [4-6] and a higher incidence of CVD than the general population [7,8].

Diabetes is well established as an important risk factor for CVD and type 2 diabetes increases the risk of coronary heart disease (CHD) by two to four folds in populations of European descent [9]. Immigrants from Asia and Africa living in Europe have a higher prevalence of diabetes than Europeans [10-12]. In the UK, CHD mortality rates among South Asians are 1.5 times that of the general population [13], while the ethnic difference in CVD mortality are three times higher among individuals with diabetes [14]. Social disadvantage is associated with CVD and most of the associated risk factors [15] and ethnic disparities in socioeconomic position (SEP) contribute to the observed ethnic inequalities in health outcomes, including CVD and diabetes [15,16].

Due to migration from Asia and Africa over recent decades, the capital of Norway (Oslo) has now become multi-ethnic, with approximately 27% of the population comprised by first and second generation ethnic minorities [17]. Previous studies of five EMGs in Oslo have revealed significant group differences in levels of serum triglycerides (TG), high-density lipoprotein (HDL)-cholesterol, blood pressure (BP), and smoking prevalence [18,19]. Furthermore, EMGs from Asia had a high prevalence of diabetes and CHD [19-21], and socioeconomic position differed between and within the various EMGs [20]. It remains unknown whether the high CVD prevalence rate among ethnic minorities in Norway is attributable to the high prevalence of diabetes.

The aim of the present study was to assess the prevalence of self-reported CVD and its associated risk factors stratified by diabetes status, and to investigate the associations between risk factors and CVD in five EMGs compared to ethnic Norwegians.

## Methods

### Participants and setting

Between 2000 and 2002, three population-based, cross-sectional studies were conducted in Oslo by The Norwegian Institute of Public Health, in collaboration with the University of Oslo, the Norwegian University of Sport and Physical Education, the Oslo Diabetes Research Centre and the Oslo Municipality. These three studies included the Oslo Health Study, the Oslo Immigrant Health Study, and the Romsås in Motion Study. These studies will be collectively referred to as the Oslo Health Studies in the present study. Although different population subgroups were targeted, the same protocol was used for all three studies (for additional details, see [18,19,21-24]).

In the Oslo Health Study, a total of 18,770 (46%) of all Oslo residents born in 1924, 1925, 1940, 1941, 1955, 1960 and 1970 participated [22]. In the Oslo Immigrant Health Study, a total of 7,607 residents born in Sri-Lanka, Pakistan, Iran, Vietnam and Turkey between the years 1942 and 1971 were invited and 39.7% (N = 3,019) participated [23]. In the Romsås in Motion Study, a total of 2,960 (48%) of all residents born between 1933 and 1969 in two districts with low SEP and a multi-ethnic population participated [24]. The Oslo Health Studies were approved by the Regional Ethics Committee South-Eastern A and The Norwegian Data Inspectorate.

In the present study, data from the Oslo Health Studies were pooled, such that the total number of invited participants was 54,473, of which 45.4% (N = 24,749) participated. We restricted our analyses to participants aged 30 to 61 years old who were born in Norway, Sri-Lanka, Pakistan, Iran, Vietnam, and Turkey between 1940 and 1971 (N = 18,523). Of these, 13,273 (71.7%) were from the Oslo Health Study, 3,019 (16.3%) from the Oslo Immigrant Health Study, and 2,231 (12.0%) from the Romsås in Motion Study. A total of 669 (3.6%) participants were excluded either due to missing data for diabetes status, which was a stratification variable (N = 563), or participation in more than one study (specifically, N = 106 individuals from the Romsås in Motion Study), leaving a final sample of 17,854 participants.

The questionnaires were translated into five languages (Tamil, Urdu, Persian, Vietnamese and Turkish) and included information on self-reported diabetes, angina pectoris (AP), myocardial infarction (MI), stroke, use of medication, health-related behaviours, education, and employment status (full- or part-time). Body height (cm), weight (kg), and waist and hip circumference (cm) were measured and body mass index (BMI; kg/m2) and waist-to-hip ratio (WHR) were calculated.

### Variables

Ethnicity was assigned by country of birth data supplied by Statistics Norway. The majority of the participants from Asia and the Middle East (aged 30 to 61 years) were first-generation immigrants when the Oslo Health Studies were conducted. Cardiovascular disease was defined as the self-reported occurrence of AP, MI, or stroke. Hypertension was defined as one of the following: systolic blood pressure (SBP) ≥ 140 mmHg, diastolic blood pressure (DBP) ≥ 90 mmHg, use of antihypertensive medications, and hypercholesterolemia defined by a total cholesterol ≥ 6.2 mmol/l, or the use of lipid-lowering medications [19]. To assess SEP, we used self-reported years of education to define early adulthood SEP and employment status (full- or part-time; yes vs. no) as a proxy for adult SEP. Body height was used as a proxy for early-life SEP [21,25,26]. Level of physical activity was categorized as sedentary if participants reported reading and watching TV as their main leisure-time activities.

### Statistical analyses

Due to dissimilar age distributions among the ethnic groups, the prevalence of CVD and diabetes with 95% confidence intervals (CIs) were standardized by age and gender using direct standardization methods with ethnic Norwegians in the study sample as the reference standard. Differences in prevalence rates between the groups, and between the particular ethnic minority group and the ethnic Norwegians were tested by likelihood ratio tests and z-tests respectively.

Chi-squared tests and one-way ANOVAs were applied to investigate differences in proportions and means, respectively, between the ethnic groups. Adjusted means and mean differences were estimated and tested by analyses of covariance (ANCOVAs). Due to a highly skewed distribution, the triglyceride values were log-transformed before applying the ANCOVA and the results were back-transformed to the original scale using anti-log.

The minority groups were pooled in some of the logistic regression analyses due to small numbers of individuals with CVD and diabetes. Adjusted odds ratios (OR; 95% CI) of having CVD by diabetes status in the minority groups compared to ethnic Norwegians were estimated by multiple logistic regression analyses.

Associations between self-reported CVD and associated risk factors were examined by logistic regression analyses. Two-way interactions between country of birth and CVD risk factors were tested by likelihood ratio tests. Two-sided tests were used and p-values ≤0.05 were considered statistically significant. The analyses were performed with SPSS 18.0 and STATA 11.0. The graphs were made in R 2.11.1 for Windows.

## Results

Of the 17,854 participants aged 30 to 61 years old, 8,371 (46.9%) were men, 13,967 (78.2%) were born in Norway, and 3,887 (21.9%) were born in Sri Lanka, Pakistan, Iran, Vietnam or Turkey. The groups differed by age, education and employment (Table 1).

**Table 1:** Baseline characteristics for participants (N = 17,854) by country of birth

*Characteristics*^a^	*Norway* *(n = 13967)*	*Sri Lanka* ***(n = 1127)***	*Pakistan* ***(n = 859)***	*Iran* ***(n = 695)***	*Vietnam* ***(n = 658)***	*Turkey* ***(n = 548)***	**p**^**b**^
**Men** % (95% CI)	44.5 (43.6-45.3)	60.0 (57.1-62.8)	54.2 (50.9-57.6)	59.8 (56.2-63.4)	45.8 (42.0-49.6)	55.3 (51.1-59.4)	< 0.001

**Age**, years Mean (95% CI)	45.2 (45.0-45.4)	39.6 (39.2-39.9)	43.3 (42.7-43.9)	41.6 (41.1-42.1)	43.2 (42.6-43.8)	41.5 (40.9-42.2)	< 0.001

**Self-reported CVD** Valid cases (n) Age at diagnosis, years Mean (95% CI)	384 46.6 (45.6-47.7)	44 46.2 (42.5-49.8)	59 48.2 (45.2-51.1)	41 44.3 (41.0-47.6)	43 43.8 (40.6-47.1)	32 47.1 (43.0-51.2)	0.335

**Self-reported DM** Valid cases (n) Age at diagnosis, years Mean (95% CI)	260 40.4 (38.8-42.0)	99 44.2 (41.6-46.8)	115 41.4 (39.1-43.8)	17 39.8 (33.0-46.5)	38 45.9 (41.3-50.4)	33 44.1 (40.0-48.3)	0.057

**Education**, years Mean (95% CI)	14.1 (14.0-14.2)	12.8 (12.5-13.0)	10.1 (9.8-10.4)	14.1 (13.8-14.5)	10.8 (10.5-11.2)	8.5 (8.0-9.0)	< 0.001

**Employment** % (95% CI)	86.4 (85.8-87.0)	77.6 (75.1-80.0)	54.0 (50.5-57.5)	66.1 (62.5-69.6)	69.0 (65.3-72.6)	57.5 (53.2-61.7)	< 0.001

^a^CVD: cardiovascular disease (i.e., angina pectoris or myocardial infarction or stroke). DM: diabetes mellitus. Employment included full-time or part-time work.

^b ^p-values. Chi-square tests and one-way ANOVAs were applied for testing differences in proportions and in means between groups, respectively. Multiple regression models were applied to compare mean age at diagnososis of CVD and diabetes between groups adjusted for age.

**Figure 1:**
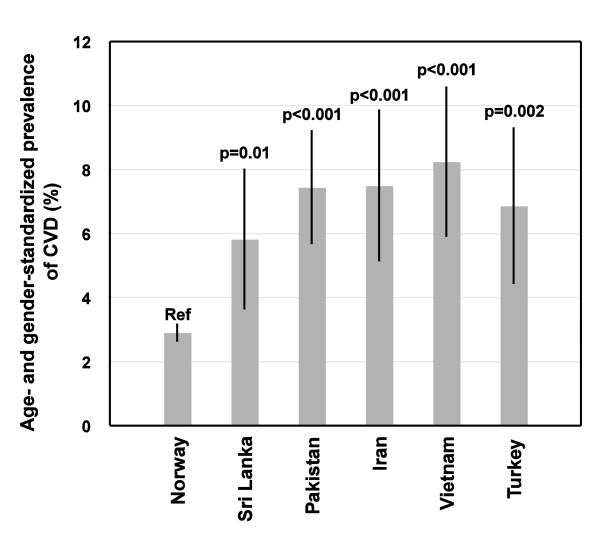
**Prevalence (% with 95% CI) of self-reported cardiovascular disease (CVD) by ethnicity**. Bars: CVD prevalence, |: 95% CI, p-value: ethnic difference in CVD prevalence between the particular ethnic group and the Norwegian reference group (ref).

Diabetes was reported by a total of 562 (3.1%) and CVD by 603 (3.4%) participants, of which 471 (2.6%) reported one CVD diagnosis (AP: 219, MI: 116, stroke: 136), 117 (0.7%) reported two diagnoses (AP and MI: 100, AP and stroke: 15, MI and stroke: 2), and 15 (0.08%) reported three CVD diagnoses. For ethnic Norwegians, 48 (12.5%) of those reporting CVD also reported diabetes, compared to 50 (22.8%) among the ethnic minorities after pooling all groups. The age- and gender-standardized prevalence rate of self-reported CVD was significantly higher for all EMGs (Sri Lanka: 5.8%, Pakistan: 7.4%, Iran: 7.5%, Vietnam: 8.2%, Turkey: 6.8%) than ethnic Norwegians (2.9%, p < 0.001 for between groups difference) (Figure 1). The EMGs also reported significantly more diabetes (age- and gender-standardized; Sri-Lanka: 13.4%, Pakistan: 15.0%, Iran: 3.0%, Vietnam: 6.7%, Turkey: 9.1% vs. Norwegians: 1.8%, p < 0.001).

**Figure 2:**
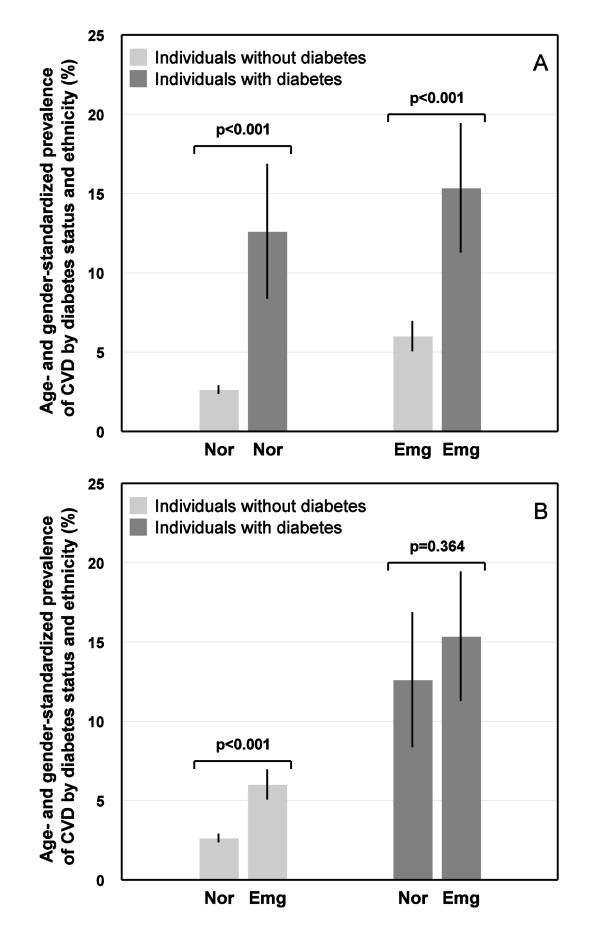
**Prevalence (% with 95% CI) of cardiovascular disease (CVD) by diabetes status and ethnicity**. Nor: Norwegians; Emg: the pooled ethnic minority group. Bars: CVD prevalence, |: 95% CI, p-value: difference in CVD prevalence between those with and without diabetes in Norwegians and the pooled ethnic minority group (A) and between Norwegians and the pooled ethnic minority group by diabetes status (B).

Individuals with diabetes reported more CVD than those without diabetes, irrespective of ethnicity (Figure 2A). The ratio between CVD prevalence for individuals with and without diabetes was 4.8 for ethnic Norwegians and 2.6 for the pooled EMG. Among individuals without diabetes, the CVD prevalence was higher for the pooled EMG compared with Norwegians (6.0% vs. 2.6%, p < 0.001) (Figure 2B). Among individuals with diabetes, the CVD prevalence for the pooled EMG was not significantly higher than ethnic Norwegians (15.3% vs.12.6%, n.s.).

For individuals without diabetes, OR for CVD adjusted for age and gender (Model 1) was higher for all EMGs (ranging from 2.1 for Sri-Lankans to 3.5 for Iranians), compared with Norwegians (p < 0.001, Table 2). After additional adjustment for adult SEP as determined by education and employment status (Model 2), and body height as a measure of early-life SEP (Model 3), the ethnic differences in OR for CVD were reduced, yet ORs in the EMGs still remained higher than ethnic Norwegians (1.5-2.6, p < 0.05), except for participants from Turkey. For individuals with diabetes, however, OR for CVD did not significantly differ between the pooled EMG and Norwegian group.

**Table 2:** Odds ratios for cardiovascular disease in individuals with and without diabetes by country of birth

			***Model 1***^b^		***Model 2***^c^		***Model 3***^d^	
*Variable*^a^	Country of birth	Valid Cases						
			***OR (95% CI)***	***P***^**e**^	***OR (95% CI)***	***P***^e^	***OR (95% CI)***	***P***^e^
CVD, Diabetes, no								
	Norway	336	Ref		Ref		Ref	
	Sri Lanka	31	2.1 (1.4-3.0)	< 0.001	1.8 (1.2-2.7)	0.003	1.7 (1.1-2.6)	0.017
	Pakistan	37	2.6 (1.8-3.7)	< 0.001	1.6 (1.1-2.4)	0.019	1.5 (1.0-2.3)	0.048
	Iran	39	3.5 (2.4-4.9)	< 0.001	2.8 (1.9-4.0)	< 0.001	2.6 (1.7-3.9)	< 0.001
	Vietnam	33	3.0 (2.0-4.4)	< 0.001	2.3 (1.6-3.5)	< 0.001	2.1 (1.4-3.4)	0.001
	Turkey	29	3.3 (2.2-5.0)	< 0.001	1.6 (1.0-2.6)	0.061	1.5 (0.9-2.5)	0.112

CVD Diabetes, yes								
	Norway	48	Ref		Ref		Ref	
	Other countries	50	1.3 (0.8-2.0)	0.378	1.2 (0.7-2.1)	0.471	1.2 (0.6-2.1)	0.729

^a ^CVD: self-reported cardiovascular disease; Diabetes, no: cases not reporting diabetes; Diabetes, yes: reported diabetes.

^b,c,d ^Logistic regression was used to estimate odds ratios (ORs) for self-reported CVD in the minority groups compared to the reference group (Norwegians). Adjusted for age and gender in Model 1. Additionally adjusted for education and employment in Model 2. Further adjusted for body height in Model 3.

^e ^p-value.

For the majority of cardiovascular risk factors, ethnic differences were observed regardless of diabetes status (Table 3). Compared to ethnic Norwegians, most EMGs had higher levels of TG, total cholesterol/HDL-cholesterol ratio, WHR, BMI, and they reported less physical activity. On the other hand, most of the EMGs had lower SBP and DBP than Norwegians. In the Norwegian and Pakistani groups, individuals with diabetes had higher SBP than those without diabetes. In nearly all ethnic groups, individuals with diabetes had higher TG, WHR, and BMI compared to individuals without diabetes. When applying the definition of obesity proposed by the WHO for Asians (BMI ≥ 25 kg/m) to participants without diabetes, 58.4% of Sri-Lankans, 76.3% of Pakistanis and 28.1% of Vietnamese were obese (values adjusted for age and gender), compared to obesity rates of 65.9%, 92.0%, and 55.8% for individuals with diabetes for these groups, respectively. Daily smoking was most prevalent among ethnic Norwegians and men from Turkey.

**Table 3:** Risk factors^a ^for cardiovascular disease in individuals with and without diabetes by country of birth

***Risk factor***^b^	*Diabetes* *status*	*Norway* *(N = 13967)*	*Sri Lanka* *(N = 1127)*	*Pakistan* *(N = 859)*	*Iran* *(N = 695)*	*Vietnam* *(N = 658)*	*Turkey* *(N = 548)*
**% CVD/DM**^c^		2.9/1.8	5.8/13.3	7.4/15.0	7.5/3.0	8.2/6.7	6.9/9.1

**BMI**, kg/m2 Mean (95% CI)	DM,no DM,yes	25.6 (25.5-25.7) 28.8 (28.3-29.3)	26.0 (25.8-26.3) 26.6 (25.8-27.4)	28.0 (27.7-28.3) 29.9 (29.2-30.7)	26.4 (26.1-26.7) 28.1 (26.1-30.0)	23.5 (23.2-23.8) 25.3 (24.0-26.6)	28.9 (28.6-29.3) 30.8 (29.4-32.2)
p =		< 0.001	0.199	< 0.001	0.092	0.010	0.012

**WHratio**, Mean (95% CI)	DM,no DM,yes	0.84 (0.84-0.85) 0.90 (0.89-0.91)	0.88 (0.88-0.89) 0.92 (0.91-0.93)	0.89 (0.89-0.90) 0.93 (0.92-0.95)	0.85 (0.84-0.85) 0.89 (0.86-0.92)	0.84 (0.83-0.84) 0.88 (0.85-0.90)	0.87 (0.86-0.87) 0.90 (0.88-0.92)
p =		<0.001	<0.001	<0.001	0.007	0.001	0.004

**SBP**, mmHg Mean (95%CI)	DM,no DM,yes	128.4(128.1-128.7) 132.1(130.2-134.1)	123.7(122.8-124.7) 126.1(123.1-129.2)	124.2(123.1-125.4) 129.7(126.9-132.5)	121.1(119.9-122.2) 124.8(117.5-132.0)	121.5(120.3-122.7) 121.1(116.2-125.9)	123.5(122.1-124.8) 127.2(122.0-132.4)
p =		< 0.001	0.136	< 0.001	0.326	0.871	0.172

**DBP**, mmHg Mean (95% CI)	DM,no DM,yes	74.9 (74.7-75.0) 74.7 (73.4-75.9)	74.3 (73.7-75.0) 74.7 (72.7-76.7)	74.5 (73.8-75.2) 75.5 (73.7-77.4)	72.1 (71.3-72.8) 73.6 (68.8-78.4)	72.8 (72.0-73.6) 73.0 (69.7-76.2)	73.2 (72.3-74.1) 73.0 (69.6-76.5)
p =		0.761	0.751	0.307	0.540	0.919	0.925

**S-Chol/** **HDL-chol ratio** Mean (95% CI)	DM,no DM,yes	4.0 (4.0-4.1) 4.3 (4.2-4.5)	4.9 (4.8-4.9) 4.8 (4.6-5.1)	4.8 (4.7-4.9) 5.1 (4.8-5.3)	4.5 (4.4-4.6) 4.6 (4.0-5.2)	4.1 (4.0-4.2) 4.3 (3.9-4.8)	4.7 (4.5-4.8) 4.9 (4.5-5.3)
p =		< 0.001	0.822	0.051	0.600	0.325	0.274

**S-TG**, mean (95% CI)	DM,no DM,yes	1.6 (1.5-1.6) 2.1 (1.8-2.4)	2.0 (1.6-2.4) 2.8 (1.3-5.8)	2.2 (1.9-2.4) 3.2 (2.5-4.2)	1.7 (1.3-2.2) 2.2 (0.8-6.1)	1.6 (1.4-1.9) 2.1 (1.2-3.8)	2.4 (2.0-2.8) 3.9 (1.9-8.2)
p =		< 0.001	0.064	< 0.001	0.948	0.382	0.001

**% Current smoker**	DM,no DM,yes	30.4 (29.6-31.1) 39.8 (32.4-47.3)	12.1 (9.3-14.9) 11.7 (5.0-18.4)	21.7 (18.5-24.9) 10.6 (4.7-16.4)	34.8 (30.8-39.0) 24.4 (4.4-44.4)	17.9 (14.7-21.0) 17.8 (4.6-30.9)	37.5 (33.0-42.0) 55.6 (35.6-75.6)
p =		0.021	0.916	0.001	0.315	0.988	0.085

**% Sedentary**	DM,no DM,yes	21.2 (20.4-21.9) 25.0 (18.6-31.4)	54.6 (50.1-59.1) 46.7 (35.4-57.9)	56.7 (52.7-60.7) 59.0 (47.8-70.3)	46.8 (42.3-51.3) 70.1 (46.9-93.3)	57.4 (53.1-61.7) 44.7 (26.9-62.6)	55.1 (50.1-60.2) 74.7 (55.2-94.1)
p =		0.238	0.200	0.704	0.053	0.178	0.056

^a ^Age- and gender- adjusted risk factors. Triglycerides were also adjusted for time since last meal before the blood sample was taken. The prevalence of smoking and sedentary behaviour were adjusted for age.

^b ^SBP: systolic blood pressure, DBP: diastolic blood pressure, S-Chol/HDL-chol ratio: S-cholesterol/HDL-cholesterol ratio, TG: triglyceride, WHR: waist-to-hip ratio, BMI: body mass index, Sedentary: reading and watching TV as main leisure-time activities.

The triglyceride variable was log-transformed before applying the multiple regression model for estimation due to highly skewed distribution of the data. The results (estimates and 95% confidence intervals) were transformed back to the original scale using anti-log.

^c^CVD/DM: age- and gender-standardized prevalence of self-reported cardiovascular disease or diabetes mellitus.

p-values. Multiple regression models were applied to estimate marginal mean values and examine differences between those with and without diabetes in the particular ethnic group, adjusted for age, gender, ethnicity and diabetes status.

The multivariate logistic regression analyses revealed that age, diabetes, hypertension, hypercholesterolemia, and WHR were significant risk factors for CVD in both the Norwegian group and the pooled EMG. Education was inversely associated with CVD and the OR for CVD was lower in women than men for ethnic Norwegians, but these associations were weaker among the pooled EMG. Ethnic differences were observed in OR for age, diabetes, and hypercholesterolemia (Table 4). Odds ratios for CVD when reporting diabetes were lower in the pooled EMG compared to Norwegians (2.2 vs. 4.4, p = 0.024).

**Table 4:** Odds ratios (OR) for cardiovascular disease (CVD) by risk factors in the ethnic groups

	*Norwegian group*	*Ethnic minority group*^*b*^	
*Risk factors*^*a*^			
	OR^c ^(95% CI)	OR^c ^(95% CI)	P^d^
Age (≥ 50 years vs. < 50 years)	4.4 (3.2-5.9)	2.4 (1.7-3.3)	0.008
Gender (women vs. men)	0.5 (0.4-0.7)	0.8 (0.5-1.2)	0.070
Self-reported diabetes (yes vs. no)	4.4 (2.9-6.6)	2.2 (1.4-3.4)	0.024
WHR (continuous)	1.2 (1.0-1.4)	1.4 (1.1-1.8)	0.276
Current smoking (yes vs. no)	1.2 (0.9-1.5)	1.3 (0.9-1.8)	0.854
Hypertension (yes vs. no)	1.9 (1.5-2.5)	1.9 (1.3-2.6)	0.899
Hypercholesterolemia (yes vs. no)	2.6 (2.0-3.3)	1.7 (1.2-2.3)	0.029
Education duration			
10-12 years vs. 0-9 years	0.8 (0.6-1.1)	0.7 (0.5-1.1)	0.773
13-15 years vs. 0-9 years	0.7 (0.5-0.9)	0.9 (0.6-1.4)	0.286
> 15 years vs. 0-9 years	0.5 (0.4-0.7)	0.8 (0.5-1.2)	0.190

^a ^Risk factors for CVD: age, gender, diabetes status, WHR = waist-to-hip ratio, Hypertension = Systolic blood pressure ≥ 140 mmHg or diastolic blood pressure ≥ 90 mmHg or use of medication for hypertension, Hypercholesterolemia = serum cholesterol ≥ 6.2 mmol/l or use of lipid-lowering medications.

^b ^Ethnic minority group: pooled group with individuals born in Sri Lanka, Pakistan, Iran, Vietnam and Turkey.

^c ^Logistic regression was applied to estimate odds ratios (OR) with 95% CI for CVD for risk factors. Each OR was adjusted for all other variables in the table.

^d ^p: significance level of the interaction between the particular risk factor and country of birth adjusted for the other variables.

## Discussion

The present study investigated differences in the prevalence of cardiovascular disease and associated risk factors by diabetes status in five ethnic minority groups living in Norway. Higher rates of cardiovascular disease were found among all ethnic minority groups compared to ethnic Norwegians, yet these differences were not related to diabetes status. Specifically, a higher prevalence of CVD in the pooled EMG was found exclusively among participants without diabetes. For these individuals, the odds ratio for CVD remained higher in most of the ethnic minority groups compared to ethnic Norwegians after adjustment for age, gender, and socio-economic position.

The high prevalence rates of cardiovascular disease and diabetes among ethnic minorities are in accordance with other Norwegian [19,21], and European studies [8,12,27]. The prevalence of self-reported diabetes among individuals from Sri-Lanka (13.4%) and Pakistan (15%) was somewhat lower than for South Asians living in the UK and US (20% and 18%, respectively) [28]. Several studies indicate that about 40-50% of diabetes cases may be undiagnosed [21,29]. We were unable to address undiagnosed diabetes in our study, and this may have contributed to the higher CVD prevalence in ethnic minorities without an established diagnosis of diabetes. The small ethnic difference in CVD prevalence among individuals reporting diabetes may indicate that a diagnosis of diabetes alerts doctors to treat CVD risk factors more actively regardless of ethnicity, following clinical guidelines that recommend regular visits to the general practitioner (GP). In line with this result, a study from Oslo found no ethnic differences in process of care for patients with type 2 diabetes treated in primary care [30]. As the immigrant population from Asia and Africa is rather young in Norway, GPs may be unaware of the greater susceptibility for CVD than diabetes among ethnic minorities, as the latter is typically more prevalent during middle age. In addition, barriers to lifestyle intervention may exist for GPs and/or ethnic minority patients. Cultural beliefs, norms, and values in certain groups (e.g., a large body size is "healthier" than a thin one, exercise is not associated with health), as well as limited economic opportunities, may fail to encourage physical activity [31]. Furthermore, many first generation ethnic minorities are exposed to a rapid transition of lifestyle characterized by an increased intake of energy dense foods and reduced need for daily physical activity, leading to obesity, dyslipidemia and thus, increased risk of CVD upon immigration [28]. Other factors, such as a higher level of psychosocial distress [32] which is not captured by the traditional risk factor assessments, may also contribute to the observation that ethnic minorities without diabetes had a less favourable risk factor profile and higher prevalence of CVD, despite lower blood pressure. Similar risk factor profile differences between ethnic minorities and the general population have been reported by others [5,33-35].

The relationships between socioeconomic position, ethnicity, and health are complex and dynamic, varying across countries and over time [36]. Nevertheless, there is growing evidence that a large part of ethnic disparities in health are a consequence of socioeconomic differences [37]. The majority of ethnic minority groups in our study were more disadvantaged than ethnic Norwegians, as indicated by lower levels of education and employment. In line with these findings, the ethnic differences in ORs for CVD among individuals without diabetes were reduced, yet remained significant following adjustment for socioeconomic position, except for the relatively small Turkish group. However, residual confounding may exist due to our reliance on rather crude measures of SEP [37].

The value of clinical measures of generalized or central obesity in the assessment of cardiovascular risk in primary prevention is not entirely clear, as national and international guidelines have provided different recommendations [38]. Among Norwegians, waist-to-hip ratio was not strongly associated with CVD and might not improve CVD risk prediction when additional information about diabetes status, hypertension, and hypercholesterolemia are available [39]. In contrast, obesity was prevalent for all EMGs and waist-to-hip ratio should be treated as a strong predictor of CHD in these groups [39,40].

A notable strength of this population-based study is the sampling technique, which was based upon the unique personal identity number assigned to all Norwegian residents. This approach arguably improves the study's representativeness and further, enabled an assessment of selection bias, as the population registries include demographic and socioeconomic data in addition to country of birth for all residents. From a public health perspective, studies that investigate and monitor health disparities along ethnic lines are warranted, owing to the rapid demographic transition over the past decades in Norway. To facilitate participation in the Oslo Health Studies, fieldworkers fluent in participants' native languages provided assistance. Our study, which included a large sample of first-generation immigrants drawn from Norway's largest ethnic minorities, enabled a comparison with ethnic Norwegians and extends prior research regarding health disparities among ethnic minorities not least in Norway, but also in Scandinavia. In particular, rather few studies have investigated the health status of Sri Lankan and Vietnamese populations in Europe.

Several limitations deserve acknowledgment. Our study is limited by a cross-sectional design, whereas a high-quality and prospective registration of cardiovascular disease would have enabled a more precise investigation of disease burden. Additionally, there were relatively low participation rates and small number of angina, MI, stroke and diabetes cases among some ethnic groups. Therefore, CVD included all three diagnoses to improve the statistical power. Nevertheless, a comprehensive analysis of the non-attendance in the Oslo Health Study concluded that prevalence estimates were robust despite considerable non-attendance [41]. This finding also applied to the ethnic minority participants. Furthermore, the five ethnic minority groups accounted for 22% of the study population, whereas ethnic minorities (first and second generation) comprised 20% in the background population in Oslo at the time of the Oslo Health Studies, providing evidence to support the study's representativeness. Although the prevalence estimates may nevertheless be prone to some selection bias, it is feasible that the associations between risk factors and outcomes within the ethnic groups would be less affected.

Self-reported chronic disease may be affected by recall and misclassification bias, possibly yielding an underestimate of disease prevalence, particularly undiagnosed or subclinical disease. Similarly, gender and ethnic differences in self-reported symptoms have been observed. For example, women and immigrants from South Asia living in the UK are more likely to report atypical angina symptoms than men and Caucasians [42,43]. Nevertheless, two studies have shown high levels of concordance between self-reported health (i.e., diabetes, AP, MI and stroke) and medical records, providing some support to the validity of this self-reported data [44,45]. Still, it is possible these findings fail to apply to ethnic minorities in Norway due to inadequate translation procedures, insensitivity of items, or other reasons [46]. However, one study specifically addressing cross-cultural validity found no evidence for marked ethnic differences in the accuracy of self-reported diabetes [47].

Due to the small number of CVD and diabetes cases, combined with the dissimilar gender distribution among different ethnic groups, we were unable to examine gender differences in the prevalence of CVD or risk factor profile. Despite the limitations of the study, however, we feel that any potential selection bias fails to explain findings of ethnic differences in the prevalence of self-reported diabetes and CVD, especially among individuals without diabetes.

Our study results have implications for health-care policy and education. Specifically, health professionals, as well as ethnic minority communities, should be aware of significant ethnic differences in the burden of cardiovascular disease, diabetes, and their associated risk profiles. In addition to bolstering existing prevention efforts, such as secondary prevention of CVD and primary prevention of CVD among diabetes patients, a need exists for better detection of undiagnosed diabetes and primary prevention of CVD among ethnic minorities. This topic is not given special attention in the recent guidelines of primary prevention of CVD [48]. In particular, the finding that women from ethnic minority groups share a similar risk for CVD as their male counterparts deserves greater awareness. Moreover, improved primary intervention of CVD using culturally sensitive methods that promote physical activity and weight reduction in minorities is essential and feasible [49]. Not least, national strategies to reduce ethnic disparities in SEP are required and may subsequently reduce ethnic differences in CVD and diabetes [37]. Longitudinal studies are needed to monitor ethnic disparities in risk profile and disease rates, and to assist with the development and evaluation of targeted preventive strategies [50].

## Conclusions

Ethnic differences in the prevalence of cardiovascular disease were most prominent for individuals without diabetes. Improved primary prevention of CVD and more intensive case-finding strategies of undiagnosed diabetes in ethnic minority groups are needed to reduce unnecessary ethnic differences in CVD in the future.

## Competing interests

The authors declare that they have no competing interests.

## Authors' contributions

ATT and LMD performed the analysis. All authors participated in discussing the results. ATT wrote the first draft of the manuscript and all authors commented on all drafts and approved the final version.

## Authors' Information

ATT, MD, is a qualified specialist family medicine (GP) and a research fellow. JS, MD, PhD, is a Professor in General Practice, Head of Department of General Practice, Institute of Health and Society, University of Oslo. LMD, MSc, is a statistician. KIB, MD, PhD, is an endocrinologist and Head of Department of Endocrinology at Oslo University Hospital, Aker and Professor of Endocrinology at Faculty of Medicine, University of Oslo. HEM, MD, PhD, is a Professor at Department of Community, Institute of Health and Society, University of Oslo. AKJ, MD, PhD, is a qualified specialist family medicine (GP), qualified specialist Public Health/Community Medicine, supervisor Family Medicine and a post doctoral research fellow and Professor at Faculty of Health Sciences, Oslo University College.

## Pre-publication history

The pre-publication history for this paper can be accessed here:

http://www.biomedcentral.com/1471-2458/11/554/prepub
